# Treatment of Angiosarcoma with Pazopanib and Paclitaxel: Results of the EVA (Evaluation of Votrient^®^ in Angiosarcoma) Phase II Trial of the German Interdisciplinary Sarcoma Group (GISG-06)

**DOI:** 10.3390/cancers13061223

**Published:** 2021-03-11

**Authors:** Daniel Pink, Dimosthenis Andreou, Sebastian Bauer, Thomas Brodowicz, Bernd Kasper, Peter Reichardt, Stephan Richter, Lars H. Lindner, Joanna Szkandera, Viktor Grünwald, Maxim Kebenko, Marietta Kirchner, Peter Hohenberger

**Affiliations:** 1 Department of Hematology, Oncology and Palliative Medicine, Helios Hospital Bad Saarow, Sarcoma Center Berlin-Brandenburg, 15526 Bad Saarow, Germany; 2 Department of Internal Medicine C, University Hospital Greifswald, 17475 Greifswald, Germany; 3 Division of Orthopedic Oncology and Sarcoma Surgery, Helios Hospital Bad Saarow, Sarcoma Center Berlin-Brandenburg, 15526 Bad Saarow, Germany; dimosthenis.andreou@helios-gesundheit.de; 4 Department of Medical Oncology, University Hospital Essen, Sarcoma Center, University of Duisburg-Essen, 45147 Essen, Germany; sebastian.bauer@uk-essen.de (S.B.); viktor.gruenwald@uk-essen.de (V.G.); 5 Department of Interal Medicine 1/Oncology, Medical University Vienna-General Hospital, 1090 Vienna, Austria; thomas.brodowicz@meduniwien.ac.at; 6 Interdisciplinary Tumor Center, Sarcoma Unit, University Medical Center Mannheim, University of Heidelberg, 68167 Mannheim, Germany; bernd.kasper@medma.uni-heidelberg.de; 7 Department of Oncology and Palliative Medicine, Helios Hospital Berlin-Buch, Sarcoma Center Berlin-Brandenburg, 13125 Berlin, Germany; peter.reichardt@helios-gesundheit.de; 8 Department of Internal Medicine I, University Hospital Carl Gustav Carus, Technical University Dresden, 01307 Dresden, Germany; stephan.richter@uniklinikum-dresden.de; 9 Department of Medicine III, University Hospital Munich, Ludwig Maximilians University, 81377 Munich, Germany; lars.lindner@med.uni-muenchen.de; 10 Clinical Division of Oncology, Department of Medicine, Medical University of Graz, 8036 Graz, Austria; joanna.szkandera@medunigraz.at; 11 Department of Hematology, Hemostasis and Oncology, Hannover Medical School, 30625 Hannover, Germany; 12 Department of Hematology and Oncology, University Hospital Schleswig-Holstein, 23538 Lübeck, Germany; maxim.kebenko@uksh.de; 13 Institute of Medical Biometry and Informatics, University of Heidelberg, 69120 Heidelberg, Germany; kirchner@imbi.uni-heidelberg.de; 14 Division of Surgical Oncology and Thoracic Surgery, University Medical Center Mannheim, University of Heidelberg, 68167 Mannheim, Germany; peter.hohenberger@umm.de

**Keywords:** angiosarcoma, paclitaxel, pazopanib, efficacy, toxicity, progression-free survival

## Abstract

**Simple Summary:**

There are very few systemic treatment options for patients with advanced angiosarcomas. We therefore examined whether combined treatment with paclitaxel and pazopanib was active and well tolerated. However, we did not meet a preplanned interim target of 6/14 patients without progression of the disease at 6 months, after which finding we stopped recruitment, having enrolled a total of 26 patients. Of the patients enrolled, 46% were progression-free at 6 months. Two patients showed a complete and seven patients a partial tumor response to treatment. The progression-free survival of patients with superficial tumors was significantly longer compared to the patients with visceral tumors. A total of 10 drug-related serious adverse effects were reported in 5 patients, including a fatal hepatic failure. The results in patients with superficial tumors appear promising. Future studies should evaluate the safety and efficacy of vascular endothelial growth factor receptor (VEGFR) and immune checkpoint inhibitors with or without paclitaxel in a randomized, multiarm setting.

**Abstract:**

We aimed to evaluate the efficacy and toxicity of paclitaxel combined with pazopanib in advanced angiosarcoma (AS). The primary end point was progression-free survival (PFS) rate at six months (PFSR6). Planned accrual was 44 patients in order to detect a PFSR6 of >55%, with an interim futility analysis of the first 14 patients. The study did not meet its predetermined interim target of 6/14 patients progression-free at 6 months. At the time of this finding, 26 patients had been enrolled between July 2014 and April 2016, resulting in an overrunning of 12 patients. After a median follow-up of 9.5 (IQR 7.7–15.4) months, PFSR6 amounted to 46%. Two patients had a complete and seven patients a partial response. Patients with superficial AS had a significantly higher PFSR6 (61% vs. 13%, *p* = 0.0247) and PFS (11.3 vs. 2.7 months, *p* < 0.0001) compared to patients with visceral AS. The median overall survival in the entire cohort was 21.6 months. A total of 10 drug-related serious adverse effects were reported in 5 patients, including a fatal hepatic failure. Although our study did not meet its primary endpoint, the median PFS of 11.6 months in patients with superficial AS appears to be promising. Taking recent reports into consideration, future studies should evaluate the safety and efficacy of VEGFR and immune checkpoint inhibitors with or without paclitaxel in a randomized, multiarm setting.

## 1. Introduction

Angiosarcomas (AS) are very rare malignant mesenchymal tumors with morphological and functional features resembling endothelial cells [1]. They account for ca. 2% of all soft tissue sarcomas, with an estimated incidence of 3/1,000,000/year [2,3]. Approximately two-thirds of AS affect the skin, most commonly of the head and neck region, but they can develop anywhere in the body [4,5]. While most tumors arise spontaneously, some AS are associated with endogenous and exogenous risk factors, mainly previous radiotherapy and chronic lymphedema [4]. Their prognosis is worse compared to most soft tissue sarcomas, with reported 5-year overall survival (OS) probabilities of 35–40% for patients with localized tumors treated with curative intent and a median survival of 8–12 months for patients with metastases [4]. The course of the disease appears to be influenced by the site of origin, with visceral AS in particular showing a poorer outcome, although it remains unclear whether this is a result of differences in tumor biology or clinical presentation [4,5].

There are very few established systemic treatment options for patients with advanced AS [6]. The weekly administration of paclitaxel, a mitotic inhibitor with additional antiangiogenic activity, was evaluated in a prospective phase II trial and achieved a 6-month progression-free survival (PFS) rate of 24% and a median OS of 8.3 months [2]. Furthermore, a potential role of inhibitors of vascular endothelial growth factor receptor (VEGFR) in the treatment of advanced AS has been suggested based on the results of in vitro studies demonstrating that AS show a distinct up-regulation of vascular-specific receptor tyrosine kinases [4,7]. However, treatment with sorafenib alone, a VEGFR and RAS tyrosine kinase inhibitor (TKI), was associated with only limited antitumor activity in pretreated AS patients and a short duration of tumor control in a phase II study from the French Sarcoma Group [8]. Pazopanib, on the other hand, has demonstrated promising results in pretreated metastatic soft tissue sarcoma [9].

We decided to evaluate the efficacy and toxicity of paclitaxel combined with pazopanib and therefore conducted a multicenter open-label phase II trial in patients with advanced AS.

## 2. Materials and Methods

### 2.1. Study Population

Patients were eligible if they were 18 years of age or older and had a histologically confirmed, unresectable, locally advanced or metastatic primary or secondary AS with a documented progression in the last 6 months prior to screening. They were required to have adequate bone marrow, cardiac, gastrointestinal, liver and renal functions, an Eastern Cooperative Oncology Group (ECOG) performance status score of ≤2 and an estimated life expectancy of >3 months. At least one measurable skin lesion or one target lesion measurable with computed tomography (CT) scans or magnetic resonance imaging (MRI) was required as per the Response Evaluation Criteria in Solid Tumors (RECIST) 1.1 [10]. Women of childbearing potential and sexually active men were required to agree to the use of adequate contraception throughout the study and for 30 days after the last dose of study drug.

Exclusion criteria included: active treatment for malignant disease other than AS; prior treatment with taxanes in the last 12 months prior to study entry; any chemotherapy or radiotherapy within 14 days before start of study medication; major surgery or trauma within 28 days prior to first dose and/or presence of any nonhealing wound, fracture, ulcer or uncontrolled infection; history or clinical evidence of central nervous system metastases or leptomeningeal sarcomatosis; evidence of active bleeding or bleeding diathesis, as well as known endobronchial lesions and/or lesions infiltrating major pulmonary vessels; pregnant or breastfeeding women.

### 2.2. Study Design. Treatment and Outcomes

This phase II trial (EudraCT number:2012-005846-39, ClinicalTrials.gov Identifier: NCT02212015) was a multicenter, open-label, prospective, single-arm study conducted at 9 sites in 2 countries. Paclitaxel was administered at a dose of 70 mg/m^2^ as a 2-h intravenous infusion on days 1, 8 and 15 of a 28-day treatment cycle, after intravenous premedication with dexamethasone, diphenhydramine and cimetidine. Standard antiemetics (mainly granisetron and ondansetron) were also recommended prior to paclitaxel administration. Pazopanib was concurrently administered at a daily dose of 800 mg to be taken orally without food at least one hour before or two hours after a meal. Patients received a total of 6 cycles of paclitaxel, unless disease progression or limiting toxicity—especially peripheral neuropathy grade 2 or higher—occurred. Pazopanib was continued beyond the 6 cycles of paclitaxel treatment, until disease progression or limiting toxicities occurred. In case of side effects under combination treatment attributable to pazopanib, paclitaxel was continued as monotherapy until the end of the 6th cycle, unless patients developed disease progression or limiting toxicities under monotherapy. The protocol specified criteria for dose reductions and delays in case of limiting toxicities.

The objective of this trial was to evaluate efficacy and safety of the experimental treatment given by a combination of pazopanib with paclitaxel for patients with advanced or metastatic angiosarcoma (AS). The primary study endpoint was PFS rate at 6 months after start of study treatment, evaluated on a predefined set of target and nontarget lesions based on the RECIST 1.1 criteria. Radiographical assessments were recommended every 8 weeks or sooner, when clinically indicated, by CT or MRI scans of the chest, abdomen, and all other tumor localizations. The diameter of skin lesions was measured clinically and documented with photographs in the patient files. The evaluation of the PFS rate at 6 months had to take place at 182 days ± 32 days after the beginning of treatment. Patients who had no available evaluation at this time and no documented CR, PR, or SD at a later point, were classified per-protocol as having PD for the purposes of the primary endpoint—a definition which led to a divergence between PFS rate at 6 months and median PFS.

Secondary endpoints were OS defined as start of therapy until death, best overall response (BOR), and toxicity according to the National Cancer Institute Common Terminology Criteria for Adverse Events (CTCAE) version 4.0. The endpoint PFS rate at 3 months was added to the statistical analysis plan prior to the final study report, in order to improve the comparability of the trial’s results to previous studies. PFS was defined as start of therapy until first PD or death, whatever came first. Two subgroup analyses were planned in the protocol for primary and secondary endpoints: superficial vs. visceral and primary vs. secondary AS.

### 2.3. Sample Size

The primary statistical analysis addressed the question whether the 6-month PFS rate was higher than 35%. The sample size was calculated to detect a 6-month PFS rate of >55%, defined as a clinically relevant success, at a one-sided significance level of *α* = 0.05 with a power of ≥80%. The study used Simon’s two-stage optimal design with a planned interim futility analysis after enrolment of the first 14 patients and a maximal sample size of 44 patients in case of proceeding to the second stage. The second stage would have been completed, if at least 6 of the first 14 patients were progression-free and alive (defined as success) at 6 months. According to the study protocol, recruitment was not stopped, however it was specified that additional patients would not be included in the interim analysis. At the time of the interim analysis, a total of 26 patients had already been enrolled, resulting in an overrunning of 12 patients. The study was closed on 31 December 2019 without any further enrolment of patients for futility reasons.

### 2.4. Statistical Analysis

Baseline demographics were summarized by median with interquartile range (IQR) or frequencies in the full analysis set (FAS) of all 26 patients. The analysis of the primary endpoint was conducted at the interim analysis for the first 14 patients, as specified by the study protocol (PP set) and for all 26 patients enrolled as FAS. To guarantee the defined significance level of *α* = 0.05 in the FAS, the method to handle overrunning by Engler and Kieser was applied [11], leading to the following amendment: if ≤17/26 successes were observed, the trial would be stopped with the conclusion that the study treatment should not be further investigated. If ≥18/26 successes were observed, the trial would be stopped with the conclusion that the study treatment should be further investigated in this histology. The point estimate for PFS rate at 6 month with 90% exact Clopper-Person confidence intervals (CI), in line with a one-sided *α* = 0.05, were provided for the PP set and the FAS.

All secondary endpoints were analyzed in the FAS with all *n* = 26 patients included after the end of study. The point estimate for PFS rate at 3 months with 95% exact Clopper-Person CI was provided. Differences in proportions between subgroups were assessed by Barnard’s exact test due to the small sample size. Analyses of OS and PFS were performed with the Kaplan–Meier method and survival distributions between subgroups were compared with the log-rank test. Descriptive statistics were used for the analysis of toxicities and BOR, while differences in proportions was assessed by exact Pearson’s chi-square test. Reported *p*-values for the secondary endpoints are interpreted descriptively and a *p*-value < 0.05 is considered statistically significant. All analyses were performed in SAS^®^ System 9.4 (SAS Inc., Cary, NC, USA).

## 3. Results

### 3.1. Baseline Demographics

Between July 2014 and April 2016, 26 AS patients were enrolled in this study. Baseline demographics are presented in Table 1. The median age at enrollment amounted to 60.5 (IQR, 48–70) years. The median time between AS diagnosis and start of treatment in this trial was 6 (IQR, 1–43) months. The majority of the patients were female (*n* = 23), had an ECOG performance status score of 0 (*n* = 20) and a superficial AS primary (*n* = 18). The rate of primary versus secondary AS was exactly balanced. Of the secondary AS, eight tumors arose in irradiated fields of previous malignancies. 69% of patients had a cutaneous angiosarcoma manifestation, 31% of patients had a visceral manifestation. Distant metastases were observed in 21 patients, the majority of which were localized in the liver (*n* = 9), the bones (*n* = 7) and the lungs (*n* = 6). Only 3 patients (12%) had received systemic chemotherapy for AS prior to study enrollment.

### 3.2. Safety and Toxicity

A total of 127 cycles of paclitaxel concurrent to pazopanib were administered. The median number of cycles amounted to 6 (IQR, 4–6 cycles), with a median of 17 (IQR, 10–18) infusions. Twenty-four patients (92%) received at least 2 cycles. Paclitaxel was discontinued due to toxicity in 3 patients (12%; 1× liver toxicity, 1× allergic reaction, 1× polyneuropathy). In 23 patients with full data available, pazopanib was administered for a median of 22 (IQR, 9–35) weeks, and was discontinued due to toxicity or withdrawal of consent in 7 patients (35%; 3× liver toxicity, 2× withdrawal of consent, 1× pneumothorax, 1× poor tolerance).

Table 2 lists all related and unrelated adverse events with a toxicity grade ≥3. A total of 10 drug-related serious adverse effects were reported in 5 patients (19%). These events were increased hepatic enzymes (*n* = 3), hepatic failure, pneumothorax, dehydration, reduced general condition, gastrointestinal bleeding, fever of unknown origin and severe neutropenia. The hepatic failure occurred in a patient with a visceral secondary AS of the liver, a medical history of myelodysplastic syndrome, and previous whole body irradiation and allogenic stem cell transplantation 2 days after of start of treatment with pazopanib and 1 application of paclitaxel. It was fatal and related to the study treatment.

### 3.3. Efficacy

The primary endpoint of PFS rate at 6 months amounted to 29% (90% CI, 10–54%) in the PP set with 14 patients and to 46% (90% CI, 29–64%) in the FAS with 26 patients. The following results are reported for the FAS.

The median follow-up was 9.5 (IQR 7.7–15.4) months. The 3-month PFS rate was 62% (95%CI, 41–80%). Patients with superficial AS had a significantly higher PFS rate at 6 months of 61% (95% CI, 35.8–82.7%), compared to 13% (95% CI, 3.2–52.7%) for patients with visceral AS (*p* = 0.0247).

**Figure 1:**
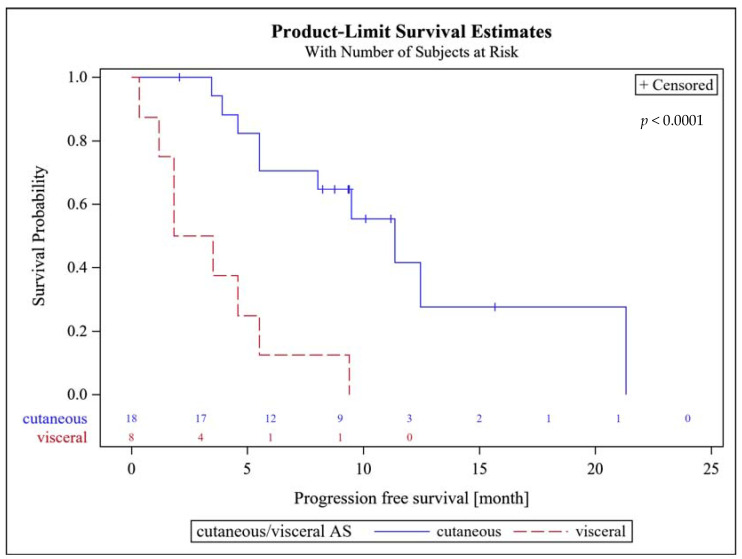
Progression free survival probability of patients with superficial (cutaneous) and visceral AS.

There was no difference in the 6-months PFS between primary and secondary AS with 46% each. The median PFS amounted to 8 (95% CI, 4.6–11.3) months. Patients with superficial AS had a significantly higher median PFS of 11.3 (95% CI, 5.5–21.1) months, compared to patients with visceral AS (2.7 (95% CI 1.2–5.5) months; *p* < 0.0001, Figure 1). There were no statistically significant differences in median PFS between patients with primary AS of 5.5 months (95% CI 3.9–12.5) and secondary AS of 9.5 months (95% CI 4.6–21.1; *p* = 0.32).

The BOR could be evaluated in all but one patient, who developed a fatal hepatic failure prior to first assessment of response. Two patients (8%) presented with CR after 4 and 5 months, seven patients (27%) had a PR after 5 to 7 months, 6 patients (23%) presented with SD and 10 patients (38%) developed progressive disease (PD). The BOR for the subgroups superficial vs. visceral and primary vs. secondary AS are presented at Table 3.

Eight deaths were observed during the follow-up period. The median OS in the entire cohort was 21.6 (95% CI, 20.5—not estimable) months. There were no statistically significant differences in median OS between patients with superficial (21.6 (95% CI, 10–21.6) months) and visceral AS (20.5 (95% CI, 2.8–not estimable) months; *p* = 0.752), or between patients with primary (20.5 (95% CI, 10–not estimable) months) and secondary AS (21.6 (95% CI not estimable) months; *p* = 0.621).

## 4. Discussion

The prognosis of patients with advanced AS is poor and only a few active systemic treatment options are available [6]. Our prospective phase II trial evaluating the efficacy and toxicity of paclitaxel combined with pazopanib did not meet its predetermined interim target of 6/14 patients progression-free at 6 months. However, at the time of this finding 26 patients had already been enrolled in the study, as a recruitment stop after completion of Simon’s stage I was not stipulated in the study protocol. The resulting overrunning necessitated an amendment of the statistical analysis plan. After a long evaluation of the feasibility of possible amendments, the study committee had to decide to terminate the trial for futility reasons at the end of 2019, as it could not be expected to meet its primary endpoint even with a modest increase of the recruitment target.

A post hoc in-depth review of the study protocol and the collected data revealed limitations of the trial design. The target group of the study was superficial AS of the extremities and scalp and secondary AS. The interim analysis had been introduced primarily under safety and toxicity aspects and the study committee expected that these patients would easily clear the threshold of 6/14 successes after 6 months. This was the reason why a recruitment stop after the first stage of the study was not included in the protocol. However, the protocol inclusion criteria did not restrict recruitment to patients with superficial AS only and several of the first 14 patients had a visceral AS, a subgroup known to have a considerably worse prognosis [4,5]. Furthermore, while previous AS studies had defined a PFS rate at 6 months of 30% [2] or 40% [12] as clinically relevant, our protocol set the bar at 55%—again based on the data on superficial AS. Thus, the early recruitment of a nonintended group of patients carrying a worse prognosis compromised the trial and led to early discontinuation for futility reasons despite the fact that the PFS rate at 6 months for the whole cohort was 46%.

It is therefore difficult to draw definitive conclusions on the role of combined paclitaxel and pazopanib treatment in AS patients. However, our results with a 6-months PFS rate in the superficial AS group of 61.1% and a median PFS of 11.6 months (Figure 1) compare favorably with the data of the EORTC STBSG group reporting a 9.5 months PFS for taxane-based therapy in superficial AS and 7 months for the overall group.

A previous phase-II trial (ANGIOTAX [2]) assessed the efficacy and toxicity of weekly paclitaxel in patients with advanced AS including visceral sites in 26% of the patients and demonstrated a median PFS of 4 months. The nonprogression rate was 74% at 2 months and 24% at 6 months. The authors concluded that the treatment regimen constitutes a good comparator for further clinical trials [2]. The follow-up study (ANGIOTAX-PLUS) was designed as a randomized, phase-II trial aiming to assess the efficacy and safety of adding bevacizumab (BWP) to weekly paclitaxel (WP) in advanced AS [12]. The median PFS amounted to 6.6 months for both arms, and there were no differences in median OS either, so that the authors concluded that both treatment arms were active but that the study results did not support additional clinical investigation of the BWP regime in patients with advanced AS [12].

Taking the results of these studies into consideration, the median PFS of 8 months observed in our trial suggests that the combination of paclitaxel with pazopanib is an active treatment of advanced AS, but it remains unclear whether the combined treatment offers advantages compared to single-agent treatment with paclitaxel. Conflicting results regarding the possible advantages of the paclitaxel/pazopanib combination compared to paclitaxel alone have previously been reported for ovarian cancer as well, with one small randomized phase-II trial demonstrating a better outcome for patients treatment with the combined regimen [13], while another small randomized phase-II trial later reported no benefit for the combined regimen [14].

An interesting finding of our analysis was that patients with superficial AS had a significantly higher median PFS of 11.3 months vs. 2.7 months for patients with visceral AS. These results confirm the findings of the ANGIOTAX-PLUS study, demonstrating a median PFS of 8 months in patients with superficial AS vs. 3.6 months in patients with visceral AS for both arms combined [12]. This did not, however, translate into significant differences in median OS between patients with superficial and visceral AS, which might be attributed to effective further treatments after progression, which were not documented in our trial. Taking these findings into consideration, we believe that future clinical trials on AS should stratify patients according to the localization of their primary tumor and perform separate analyses of the results of patients with superficial and visceral AS.

Interestingly, while the ANGIOTAX-PLUS study did not perform subgroup analyses for median OS, it did report a median OS of 19.5 months for the WP regimen, which was considerably longer compared to a median OS of 8 months for the WP regimen in the ANGIOTAX study [2,12]. This finding underlines the critical role of randomization using a concurrent internal control arm in rare diseases [12], and illustrates why it is not prudent to compare the PFS and OS rates achieved in our trial with those reported for previous studies.

In terms of toxicity of the combined paclitaxel and pazopanib regimen, only 19% of the patients in our trial developed serious adverse effects, compared to 32% of the patients treated with BWP in the ANGIOTAX-PLUS study developing drug-related serious adverse effects and no patients treated with WP [12]. Furthermore, we were able to administer slightly more paclitaxel infusions (median, 17), compared to the WP arm (median, 16) and the BWP arm (median, 14) of the ANGIOTAX-PLUS study [12]. The duration of treatment with pazopanib in our study was also slightly longer compared to the duration of bevacizumab treatment in the ANGIOTAX-PLUS study (22 vs. 18 weeks) [12]. Treatment delays and treatment discontinuation due to toxicity are important aspects of phase-II trials, as PFS and OS often depend on the dose intensity and cumulative dose achieved.

## 5. Conclusions

In conclusion, our analysis does not allow any definitive conclusions on the efficacy of combined treatment of paclitaxel and pazopanib in patients with advanced AS, although the median PFS of 11.6 months in patients with superficial AS in our study appears to be promising. On the other hand, recent case series have suggested that immune checkpoint inhibitors may be active in the treatment of advanced AS [15], while the combination of VEGFR and checkpoint inhibitors, already established in the treatment of several solid tumors, also appears to be a promising treatment option in soft tissue sarcoma patients in general [16]. We therefore believe that future studies should evaluate the safety and efficacy of VEGFR and immune checkpoint inhibitors with or without paclitaxel in a randomized, multiarm setting, as is the case in the currently recruiting NCT04339738 trial [17].

## Figures and Tables

**Table 1:** Baseline demographics and disease characteristics.

Variable	*n*	%
Study cohort	26	100%
Sex		
Female	23	88%
Male	3	12%
ECOG performance status score		
0	20	77%
1	5	19%
Not available	1	4%
Tumor site		
Superficial AS	18	69%
Visceral AS	8	31%
Tumor origin		
Primary AS	13	50%
Secondary AS	13	50%
Disease status at presentation		
Locally advanced	5	19%
Metastatic	21	81%
Liver	9	35%
Bone	7	27%
Lung	6	23%
Lymph nodes	3	12%
Other	9	35%
Prior treatments		
Surgery	14	54%
Radiotherapy	3	12%
Chemotherapy	3	12%
No prior treatments	11	42%

ECOG, Eastern Cooperative Oncology Group; AS, angiosarcoma.

**Table 2:** All adverse events (AE) grade ≥3.

Toxicity	Grade III/IV		Grade V	
	AE	Affected Patients (%)	AE	Affected Patients (%)
Increased alanine aminotransferase	11	3 (12%)	0	0 (0%)
Increased aspartate aminotransferase	3	1 (4%)	0	0 (0%)
Allergic reaction	1	1 (4%)	0	0 (0%)
Reduced general condition	2	2 (8%)	0	0 (0%)
Anemia	1	1 (4%)	0	0 (0%)
Arterial hypertension	7	2 (8%)	0	0 (0%)
Dehydration	2	1 (4%)	0	0 (0%)
Increased gamma-glutamyl transferase	6	1 (4%)	0	0 (0%)
Hepatic failure	0	0	1	1 (4%)
Anorexia	1	1 (4%)	0	0 (0%)
Catheter-related infection	1	1 (4%)	0	0 (0%)
Leukopenia	20	3 (12%)	0	0 (0%)
Fatigue	5	2 (8%)	0	0 (0%)
Neutropenia	16	3 (12%)	0	0 (0%)
Pneumothorax	1	1 (4%)	0	0 (0%)
Pleuritic pain	1	1 (4%)	0	0 (0%)
Back pain	5	2 (8%)	0	0 (0%)

**Table 3:** Best overall response in absolute and relative (%) frequencies for subgroup analyses.

**Best Overall Response (BOR)**	**Superficial AS, *n* = 18**	**Visceral AS, *n* = 8**
CR	2 (11%)	0 (0%)
PR	6 (34%)	1 (12.5%)
SD	4 (22%)	2 (25%)
PD	6 (33%)	4 (50%)
n.e.	0 (0%)	1 (12.5%)
**Best Overall Response (BOR)**	**Primary AS, *n* = 13**	**Secondary AS, *n* = 13**
CR	0 (0%)	2 (15%)
PR	5 (38.5%)	2 (15%)
SD	3 (23%)	3 (23%)
PD	5 (38.5%)	5 (39%)
n.e.	0 (0%)	1 (8%)

CR, complete response; PR, partial response; SD, stable disease; PD, progressive disease; n.e., not evaluated.

## Data Availability

The data presented in this study are available on request from the corresponding author. The data are not publicly available, in accordance with the study protocol.
